# Pure Duplication of the Distal Long Arm of Chromosome 15 with Ebstein Anomaly and Clavicular Anomaly

**DOI:** 10.1155/2011/898706

**Published:** 2011-11-13

**Authors:** Rachel O'Connor, Amel Al-Murrani, Salim Aftimos, Philip Asquith, Roberto Mazzaschi, Dominique Eyrolle-Guignot, Alice M. George, Donald R. Love

**Affiliations:** ^1^Diagnostic Genetics, LabPlus, Auckland City Hospital, P.O. Box 110031, Auckland 1148, New Zealand; ^2^Northern Regional Genetic Service, Auckland City Hospital, Private Bag 92024, Auckland 1142, New Zealand; ^3^Maternité Andrea De Balmain, Centre Hospitalier du Taaone, BP 1640, 98 713 Papeete, Polynésie Française, France; ^4^School of Biological Sciences, University of Auckland, Private Bag 92019, Auckland 1142, New Zealand

## Abstract

This report is of a patient with pure trisomy of 15q24-qter who presents with the rare Ebstein anomaly and a previously unreported skeletal anomaly. Chromosome microarray analysis allowed high-resolution identification of the extent of the trisomy and provided a means of achieving higher-resolution breakpoint data. The phenotypic expression of unbalanced chromosomal regions is a complex phenomenon, and fine mapping of the involved region, as described here, is only a first step on the path to its full understanding. Overexpression of the *LINGO-1* and *CSPG4* genes has been implicated in developmental delay seen in other patients with trisomy of 15q24-qter, but our patient is currently too young to ascertain developmental progress. The genetic underpinning of Ebstein anomaly and the skeletal anomaly reported here is unclear based on our high-resolution dosage mapping.

## 1. Introduction

Reported cases of duplication of distal chromosome 15 are uncommon. Most of these are due to the inheritance of an unbalanced product of a translocation with an additional monosomic component. There are only very few reported cases of “pure” duplication of 15q24-qter region (Table 1) [1–6]. Of the 10 reported patients, seven patients had trisomy of 15q25/q26-qter. These patients presented with evidence of prenatal overgrowth and developed postnatal overgrowth. Overgrowth has been attributed to overexpression of the insulin-like growth factor (*IGF1R*) gene, which is located in 15q26.3 and is known to play a critical role in the somatic growth of the mammalian embryo and fetus and in postnatal growth. In general, these patients also had mild developmental problems and minimal dysmorphism, the latter encompassing abnormal slant or size of the palpebral fissures, ptosis, long fingers, and overlapping toes.

In the three remaining patients, the trisomy involved the 15q24-qter region [2, 6]. Interestingly, these patients exhibited growth retardation, together with more significant developmental delay compared to those patients described above. It is tempting to speculate that overexpression of genes that lie in the 15q24 region rule out the effect of increased expression of IGF1R, leading to short instead of tall stature.

In this report we present a patient with a pure duplication of 15q24-qter. Our patient has unusual phenotypes that are not commonly associated with previously reported dup15q patients. Among her many dysmorphic findings are two atypical features. The first is Ebstein anomaly, which has been reported in only two other cases that carry trisomy for 15q22/q24-qter, but these patients also carried monosomic regions [6]. Ebstein anomaly is a distinctive abnormality of the leaflets of the tricuspid valve in which deformed septal and posterior valve leaflets are displaced apically and adherent to the wall of the right ventricle. The second atypical feature is an unusual radiologic appearance of the clavicles.

## 2. Clinical Report

The propositus was born prematurely at 33 weeks gestation to a G2, P1 mother. Her first pregnancy resulted in an early miscarriage. Antenatal diagnosis was initially undertaken at 23 weeks of gestation, and an atrial septal defect (3-4 mm) was detected, with right atrial dilatation and pulmonary hypoplasia, together with hepatomegaly and a large gall bladder; Ebstein's anomaly was diagnosed by a cardiologist. There was no evidence of growth retardation. Followup at 31 weeks gestation showed aggravation of the heart defect with right ventricular dilatation.

The birth weight was 2380 gms, length 47 cms and head circumference was 32.5 cms, which were all adequate for gestational age. Postnatal ultrasound on the first day of life confirmed the presence of a severe dysplastic tricuspid valve, with hypoplastic and thickened septal leaflet and thickened anterior leaflet. These findings were associated with the presence of severe tricuspid regurgitation. The right atrium was severely dilated, and an atrial septal defect measuring 3-4 mm in diameter was present with right to left shunting. The pulmonary annulus was slightly hypoplastic. Moderate right ventricular dilatation was also noted. On examination, small palpebral fissures and mild micrognathia were present. The fingers were long and slender and there was bilateral fifth finger clinodactyly. There was also incurving and overlapping of the toes. The scrotum was underdeveloped, the testes were undescended, and there was glandular hypospadias with associated chordee.

**Figure 1:**
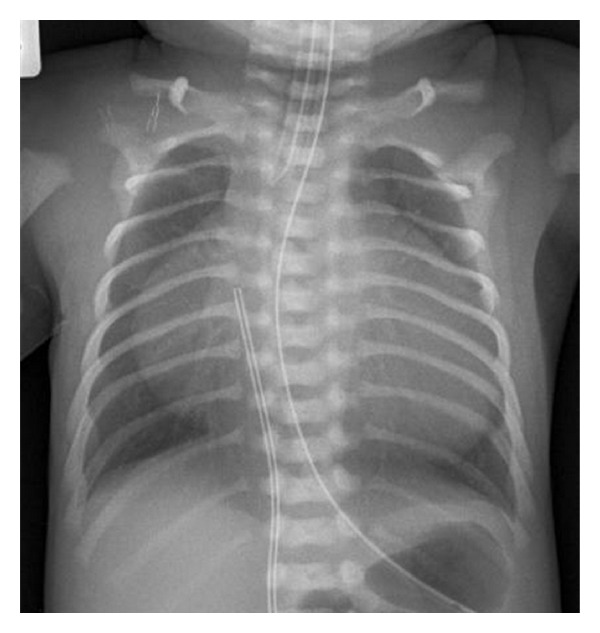
Proband's chest film on day 1 of life. This film shows massive cardiomegaly, thin and wavy upper ribs, and the bilateral anomalous clavicles.

The initial chest film revealed the presence of massive cardiomegaly with a cardiothoracic ratio of 0.83. Also noted were some skeletal anomalies (Figure 1). There were 11 pairs of ribs, and some ribs exhibited a thin and wavy appearance. Repeated chest films also demonstrated an unusual appearance of the clavicles that was likely to be caused by angular distortion of their midportions. Pseudarthrosis of the clavicles was ruled out by obtaining views of the clavicles with the arms down and up, and no pseudarthrosis could be documented. Furthermore, no masses were palpated clinically at the clavicular sites.

The infant required nasogastric feeding initially. He made slow progress, but by five weeks of age he was feeding fully orally and was gaining in weight. There were no signs of cardiac failure and his oxygen saturation readings were above 95%. Cardiomegaly settled, although the heart remained slightly enlarged on repeat radiology. A followup echocardiogram revealed right atrial dilatation with significant tricuspid regurgitation. Right ventricular function was normal and there was mild pulmonic stenosis. The atrial septal defect now had predominantly left to right shunting through it. Surgical cardiac intervention was deemed not to be required at that stage but the infant will be followed-up by a cardiologist.

### 2.1. Cytogenetic and Molecular Studies

**Figure 2:**
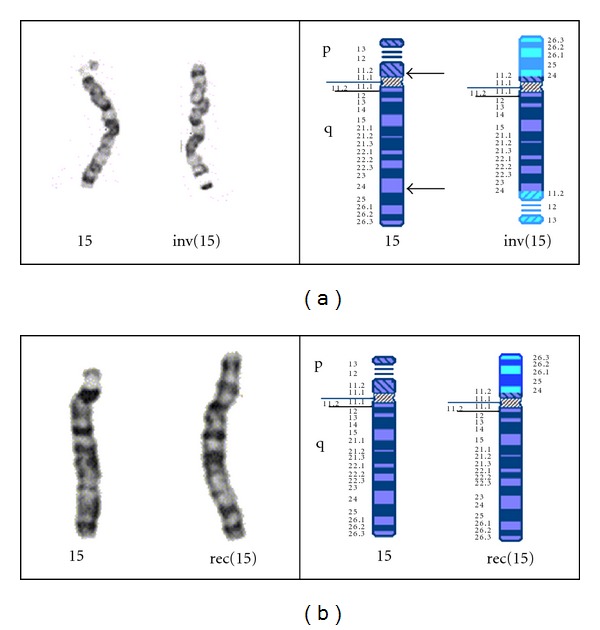
Partial karyotype and ideograms of chromosomes 15 of proband and mother. (a) The mother's partial karyotype and ideogram shows a normal chromosome 15 (left) and inverted chromosome 15 (right). (b) The proband's partial karyotype and ideogram shows the normal chromosome 15 (left) and recombinant chromosome 15 (right).

Conventional G-banded chromosome analysis was performed on peripheral blood samples taken from the proband and her mother (a blood sample from her father was not available). Analysis of the mother identified a pericentric inversion in one homologue of chromosome 15, with breakpoints at 15p11.2 and q24: 46,XX,inv(15)(p11.2q24) (Figure 2(a)). Analysis of the proband revealed additional chromosomal material on distal 15p, with a karyotype of 46,XY,rec(15)dup(15q)inv(15)(p11.2q24)mat (Figure 2(b)).

**Figure 3:**
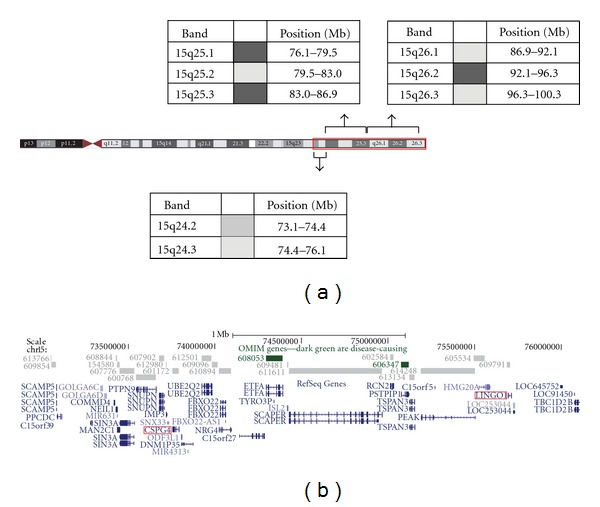
Schematic of the chromosome 15 region that shows increased dosage in the proband. (a) Shows an ideogram of chromosome 15, together with the location and extent of the duplication detected in the proband (15q24.2-qter; boxed in red); the base pair coordinates of the light and dark staining G-bands are also shown. (b) Shows the Refseq genes that are localised to 15q24.2-15q24.3; *LINGO-1* and *CSPG4* are shown in red boxes. These data and graphics were taken from the UCSC genome browser (http://www.genome.ucsc.edu).

Genomic DNA was isolated from peripheral blood of the proband using the Gentra Puregene blood kit according to the manufacturer's instructions. 0.1 micrograms of genomic DNA was labelled using the Affymetrix Cytogenetics Reagent Kit, and labelled DNA was applied to an Affymetrix Cytogenetics Array (2.7 million probes) according to the manufacturer's instructions. The array was scanned and the data was analysed using the Affymetrix Chromosome Analysis Suite (ChAS; version 1.0.1) and interpreted with the aid of UCSC genome browser (hg18 assembly). The proband's molecular karyotype identified a terminal duplication of 27 Mb; arr 15q24.2q26.3(73,237,973-100,215,737)x3 (Figure 3). 

## 3. Discussion

The patient described in this report exhibited normal growth parameters at birth, but it is too early to assess postnatal growth and developmental progress. Some of the noted dysmorphic findings, namely the small palpebral fissures, micrognathia, long slender fingers, incurving and overlapping of toes, and hypoplastic genitalia, are similar to those previously reported in patients with pure 15q24-qter duplications. The more significant developmental delay reported for these patients, compared to those with a more distal duplication (15q25/26-qter), may be due to the overexpression of genes located in the 15q24 region [7]. Of relevance are the genes *LINGO-1* and *CSPG4*. Overexpression of *LINGO-1* has been implicated in delayed myelination in mouse models [8, 9], and cells expressing CSPG4 play a role in brain repair [10, 11].

Our patient exhibits Ebstein anomaly, which is uncommon, comprising only 0.5% of all patients with a congenital heart defect [12]. The formation and liberation of the tricuspid valve leaflets occurs by a process of delamination from the underlying myocardium, starting at 8 weeks of fetal gestation. Ebstein anomaly is linked to a deficiency in this delamination process of the septal and posterior tricuspid valve leaflets [13]. The cases associated with chromosomal abnormalities, as well as the multiple cases of familial Ebstein anomaly, support the hypothesis that several genes may act in the regulation of delamination during cardiogenesis. To date, only two human genes have been linked to Ebstein anomaly: *TBX5* at 12q24.1, which is associated with Holt-Oram syndrome [14], and the cardiac transcription factor, *NKX2-5*, at 5q34 [15].

There are only two reported cases of patients with duplication of distal 15q and Ebstein anomaly (Table 1) [6]. The first patient was trisomic for the 15q22-qter region and monosomic for 7q36-qter. The second patient was trisomic for the 15q24-qter region and monosomic for 21q22.2 region. No previous cases of Ebstein anomaly have been reported in patients with terminal 7q or 21q monosomies. Thus, these two cases, together with the present case, raise the possibility that overexpression of a gene, likely within the 15q24 band, disrupts normal development of the tricuspid valve. The identity of this gene is unclear based on a search of all the genes within 15q24.2-q24.3 (see supplementary Table of Supplementary Material available at doi:10.1155/2011/898706).

The described skeletal anomalies of our case have not been previously reported in patients with partial trisomy 15q. Of particular interest is the unusual radiologic appearance of the clavicles. A similar appearance of the midportions of the clavicles has been previously reported in an infant with mosaicism of ring 21 [16]. A subsequent letter to the journal's editor raised the possibility that this anomaly may represent pseudarthrosis of the clavicles [17]. We have ruled out this possibility both clinically and radiologically in this case. Unfortunately, there are no known human genes in the 15q24-q26.3 region that appear to be involved in the development of the unusual phenotypes presented here.

## Supplementary Material

The over-expression of genes within the distal 15q24 region may play a role in the phenotype of the case described here. Those genes located within 15q24.2-15q24.3 are shown in the table below. Those highlighted in green play a role in neurological development. The phenotypic comments are taken from entries for each gene in the Online Mendelian Inheritance in Man (OMIM) web site http://www.ncbi.nlm.nih.gov/omim.

## Figures and Tables

**Table 1:** Summary of cases with duplications in the 15q24-qter region.

	Present case	Okubo et al.	Roggenbuck et al.			Bonati et al.	Kant et al.	Tatton-Brown et al.				Miller et al.	
			Twin1	Twin2				Family A		Family B			
			Case 1*	Case 2	Case 3		Case A	Case 1A	Case 1-B	Case 2-A	Case 2-B	Case 1	Case 2
Sex	F	M	F	F	M	M	F	M	F	F	M	F	F
Trisomic segment	15q24.2-q26.3	15q25-qter	15q24-q26.3	15q24-26.3	15q24-q26.3	15q25.2-qter	15q26.1-qter	15q26.1-qter	15q26.1-qter	15q26-qter	15q26-qter	15q22-qter	15q24-qter
Monosomic segment	15p11.2	15p11.2	none	none	none	none	none	15p12-pter	15p12-pter	14p11.2-pter	14p11.2-pter	7q36-qter	21q22.2-qter

Dysmorphic features													
Postnatal growth retardation		−		+	+	−	−	−	−	−	−	−	+
Tall stature		+	−	−	−	+	+	+	+	+	+	−	−
Mental retardation/Developmental delay		+		+	+	+	+	+	+	+	+	+	+
Micrognathia	+			+	−	−			−				
Long-tapered fingers	+			+	−	−		+	−			+	+
Clinodactyly	+			+	+	−			−	+			
Incurving and overlapping toes	+			+		−		+		+		+	
Unusual palpebral fissures	Small			Upslanting	Down-slanting	−			Down-slanting				Down-slanting
Hypoplastic genitalia	+			+	−	−			−				
Cardiac anomaly	Ebstein anomaly			Ventricular septation	VSD+ASD							Ebstein anomaly	Ebstein anomaly

Other	Radiologic appearance of the clavicles		Anencephaly	Microcephaly	Brachycephaly	Seizure	Wide nasal bridge	Frontal bossing	Horseshoe kidney	Strabismus Long prominent nose	Dolichocephalic	Microcephaly	Prominent nose
			*Died soon after birth	High arched palate	Ptosis	Strabismus	Long face	Prominent chin	Broad nasal bridge	Long chin	Prominent nose and chin	Prominent nose	Broad nasal bridge
				Ptosis	Blepharoptosis		Hypotonia		Large chin			Broad nasal bridge	
				Seizures	Prominent nasal bridge							Multiple joint contractures	

M: male; F: female; +: feature present; −: feature absent; blank: not known; VSD: ventral septal defect; ASD: atrial septal defect.
