# Supplementary material for: Pure Duplication of the Distal Long Arm of Chromosome 15 with Ebstein Anomaly and Clavicular Anomaly
Source: Case Rep Genet. 2011 Nov 13;2011:898706. doi: 10.1155/2011/898706 (PMC3447219; doi:10.1155/2011/898706)
Supplement: Supplementary file 1 — The over-expression of genes within the distal 15q24 region may play a role in the phenotype of the case described here. Those genes located within 15q24.2-15q24.3 are shown in the table below. Those highlighted in green play a role in neurological development. The phenotypic comments are taken from entries for each gene in the Online Mendelian Inheritance in Man (OMIM) web site http://www.ncbi.nlm.nih.gov/omim. [file 898706.f1.doc]

**Supplementary Table**

**Genes located in 15q24.2-15q24.3**

| **OMIM** | **Protein name** | **Phenotype** |
| --- | --- | --- |
| 609854 | PHOSPHOPANTOTHENOYLCYSTEINE DECARBOXYLASE; PPCDC | PPCDC functions within the CoA synthetic pathway. |
| 610288 | GOLGI AUTOANTIGEN, GOLGIN SUBFAMILY A, 6; GOLGA6 | GOLGA6 is expressed exclusively within the seminiferous tubules at specific stages of germ cell differentiation. |
| 608844 | ENDONUCLEASE VIII-LIKE 1; NEIL1 | NEIL1 belongs to a class of DNA glycosylases that initiate the first step in base excision. |
| 154580 | MANNOSIDASE, ALPHA, CLASS 2C, MEMBER 1; MAN2C1 |  |
| 607776 | SIN3, YEAST, HOMOLOG OF, A; SIN3A | Protein complexes containing SIN3A act as histone deacetylase. |
| 600768 | PROTEIN-TYROSINE PHOSPHATASE, NONRECEPTOR-TYPE, 9; PTPN9 | PTPN9 may participate in the transfer of hydrophobic ligands. |
| 607902 | RNA, U TRANSPORTER 1; RNUT1 | This protein functions as an snRNP-specific nuclear import receptor. |
| 612980 | IMP3, S. CEREVISIAE, HOMOLOG OF; IMP3 | IMP3 forms a ternary complex that interacts with U3 small nucleolar RNA (snoRNA), which is required for the early cleavage steps in pre-rRNA processing. |
| 601172 | CHONDROITIN SULFATE PROTEOGLYCAN 4; CSPG4 | CSPG4 plays a role in cell growth control, adhesion, cell-substratum interactions, and cell-cell contacts. A link has been suggested between cells expressing CSPG4 and oligodendrocytes. |
| 612501 | UBIQUITIN-CONJUGATING ENZYME E2Q 2; UBE2Q2 | UBE2Q2 can covalently bind ubiquitin. |
| 609096 | F-BOX ONLY PROTEIN 22; FBXO22 | FBXO22 acts as a protein-ubiquitin ligase. |
| 610894 | NEUREGULIN 4; NRG4 | NRG4 initiated cell-to-cell signaling through tyrosine phosphorylation. |
| 608053 | ELECTRON TRANSFER FLAVOPROTEIN, ALPHA POLYPEPTIDE; ETFA | An autosomal recessive disorder of fatty acid, amino acid, and choline metabolism. |
| 231680 | MULTIPLE ACYL-CoA DEHYDROGENASE DEFICIENCY; MADD |
| 609481 | ISL2 TRANSCRIPTION FACTOR, LIM/HOMEODOMAIN; ISL2 | An early phase of Isl2 expression by prospective mouse visceral motor neurons of the sympathetic preganglionic motor column is critical for the emergence of complete visceral motor neuron. |
| 611611 | S-PHASE CYCLIN A-ASSOCIATED PROTEIN IN THE ENDOPLASMIC RETICULUM; SCAPER | SCAPER represents a novel cyclin A/Cdk2 regulatory protein that transiently maintains cyclin A in the cytoplasm. |
| 602584 | RETICULOCALBIN 2; RCN2 | RCN2 is a calcium-binding protein, and in the mouse is a 'vitamin D receptor-associated factor'. |
| 604416 | PYOGENIC STERILE ARTHRITIS, PYODERMA GANGRENOSUM, AND ACNE | Misense mutations in this gene are implicated in an autosomal dominant disorder. |
| 606347 | PROLINE/SERINE/THREONINE PHOSPHATASE-INTERACTING PROTEIN 1; PSTPIP1 |
| 605534 | HIGH MOBILITY GROUP PROTEIN 20A; HMG20A | High mobility group (HMG) proteins are nonhistone chromosomal proteins that contain a conserved DNA-binding domain called the HMG box. |
| 609791 | LEUCINE-RICH REPEAT PROTEIN, NEURONAL, 6A; LRRN6A | Overexpression of Lingo1 leads to RhoA activation and inhibits oligodendrocyte differentiation and myelination. |

The genes highlighted in green are those that play a role in neurological development. The phenotypic comments are taken from entries for each gene in the Online Mendelian Inheritance in Man (OMIM) web site (<http://www.ncbi.nlm.nih.gov/omim>).
